# Dental Society of the State of New York

**Published:** 1883-05

**Authors:** 


### DENTAL SOCIETY OF THE STATE OF NEW YORK.

The fifteenth annual meeting of the New York State Dental Society was held in Albany, May 9th and 10th inst. There was more than the ordinary number of members in attendance, and the proceedings were of unusual interest. As the society is a representative body, made up in great part of delegates from each of the eight subordinate district societies, all of which must duly report to the parent organization, very much of the time is necessarily taken up by business. But there was abundant opportunity for the reading and discussion of several excellent papers, and for the consideration of many things of moment to the profession.

The Board of Censors met on Monday, the 8th, and had concluded their examination of candidates for the State diploma and degree, before the opening of the meeting proper. The regular sessions of the Society began in Geological Hall, at ten o'clock, A. M., of Wednesday, the 9th. Dr. L. S. Straw, of Newburg, President, in the chair. The first session was principally devoted to routine business, the reading of statements from the district societies, the reception of new delegates, and reports of committees. Dr. Charles Miller, of New York, and Dr. LeGrand Ames, of Albany, were elected permanent members. The President read the annual address, which was referred to a special committee. A committee was also appointed to report resolutions expressive of the sorrow of the members over the death of Dr. Marshall H. Webb, an honorary member. The report of the Treasurer showed a balance of moneys on hand of nearly a thousand dollars; rather an unusual exhibit for such societies.

Dr. N. W. Kingsley, Chairman of the Board of Censors, presented the report from that body. At the meeting held the day previous all the censors were present, and nine candidates for the diploma and degree of the society were examined. Of this number three had successfully passed the ordeal; namely, J. Howard Reed, of New York, John E. Taggart, of Westport, and W. C. Hayes, of Buffalo.

The afternoon session was chiefly devoted to the reading of papers. It was opened by Prof. Wm. Hailes, Jr., of Albany, who, by request of the society, presented a series of very beautiful histological preparations for the microscope. Prof. Hailes has made like exhibitions at previous meetings, and has always been warmly received, while his microscopical objects have received unstinted commendation.

Dr. S. B. Palmer, of Syracuse, read a thoughtful paper on "Professional Attainments and Popular Needs," and he was followed by Dr. A. P. Southwick, of Buffalo, whose subject was

"Cleft Palate." The discussion of these papers occupied the time until the hour for adjournment.

At the evening session three papers were read: "Longitudinal Grooves in Teeth," by C. E. Francis, of New York, "Certain Microscopical Elements in Pulpless and Gum-Denuded Teeth," by J. Edward Line, of Rochester, and "Disease of the Antrum," by Frank Abbott, of New York.

At the opening of the morning session of Thursday, Dr. C. F. W. Bodecker and Dr. N. W. Kingsley, of New York, related very interesting incidents of office practice, after which Dr. W. H. Atkinson presented a paper upon "Disease."

"Artificial Crowns" was the next subject, and Dr. N. W. Kingsley opened the discussion by describing crowns of various kinds which he had been in the habit of using. He was followed by Dr. W. Storer How, of Philadelphia, and the discussion soon became general.

The only paper presented for the Whitney memorial prize was one upon "The Extraction of Deciduous Teeth," by Dr. N. W. Kingsley. The committee recommended that although there was no competition, yet in accordance with established precedent the prize of thirty dollars be awarded the author. The paper was not read.

Dr. C. A. Woodward, of New York, C. W. Harvey, of Brooklyn, and J. Edward Line, of Rochester, were elected permanent members.

At the annual election for officers the following were chosen:

President—L. S. Straw, of Newburg.
Vice President—Wm. Jarvie, Jr., of Brooklyn.
Secretary—J. Edward Line, of Rochester.
Treasurer—H. G. Mirick, of Brooklyn.
Correspondent—W. H. Atkinson, of New York.

CENSORS.

1st dist.—N. W. Kingsley, New York.
2d dist.—Wm. Jarvie, Jr., Brooklyn.
3d dist.—S. D. French, Troy.

4th dist.—W. H. Colgrove, Johnstown.
5th dist.—S. B. Palmer, Syracuse.
6th dist.—A. M. Holmes, Morrisville.
7th dist.—Frank French, Rochester.
8th dist.—A. P. Southwick, Buffalo.

After the election of officers was completed, the society adjourned.

We regret exceedingly that we can do no more than to present such a meagre and unsatisfactory report of so important a meeting as that of the dentists of the Empire State. But the arrangements for the publication of the papers and discussions have heretofore been of such a character as to forbid their use by the journals of the profession, while the transactions were so late in making their appearance that it was "stale matter" before it could be presented, and hence we had made no arrangements for a full report. We hope that a different condition of affairs will prevail in the future, and that we may yet be enabled to lay before our readers some of the very excellent papers read at the meeting.

